# Breeding for Climate Change Resilience: A Case Study of Loblolly Pine (*Pinus taeda* L.) in North America

**DOI:** 10.3389/fpls.2021.606908

**Published:** 2021-04-30

**Authors:** Lilian P. Matallana-Ramirez, Ross W. Whetten, Georgina M. Sanchez, Kitt G. Payn

**Affiliations:** ^1^Department of Forestry and Environmental Resources, North Carolina State University, Raleigh, Raleigh, NC, United States; ^2^Center for Geospatial Analytics, North Carolina State University, Raleigh, Raleigh, NC, United States

**Keywords:** abiotic stress, drought, cold, loblolly pine, tree breeding, tree physiology, conifer genomics, climate change

## Abstract

Earth’s atmosphere is warming and the effects of climate change are becoming evident. A key observation is that both the average levels and the variability of temperature and precipitation are changing. Information and data from new technologies are developing in parallel to provide multidisciplinary opportunities to address and overcome the consequences of these changes in forest ecosystems. Changes in temperature and water availability impose multidimensional environmental constraints that trigger changes from the molecular to the forest stand level. These can represent a threat for the normal development of the tree from early seedling recruitment to adulthood both through direct mortality, and by increasing susceptibility to pathogens, insect attack, and fire damage. This review summarizes the strengths and shortcomings of previous work in the areas of genetic variation related to cold and drought stress in forest species with particular emphasis on loblolly pine (*Pinus taeda* L.), the most-planted tree species in North America. We describe and discuss the implementation of management and breeding strategies to increase resilience and adaptation, and discuss how new technologies in the areas of engineering and genomics are shaping the future of phenotype-genotype studies. Lessons learned from the study of species important in intensively-managed forest ecosystems may also prove to be of value in helping less-intensively managed forest ecosystems adapt to climate change, thereby increasing the sustainability and resilience of forestlands for the future.

## Introduction

Plants experience stress when they receive any type of stimulus that disrupts metabolic homeostasis and affects growth, development, and productivity. During the first phases of the stress event, the plant will use energy to find a new equilibrium by adjusting metabolic pathways, thereby increasing the probability of survival in a process that is usually referred to as acclimation or physiological adaptation.

Two general mechanisms of dealing with stress are avoidance and tolerance. Avoidance implies that plants evade the stressful condition by preventing or reducing the exposure to its deleterious effects, whereas tolerance consists of responses that enable plants to endure or withstand the adverse conditions (McDowell et al., 2008; Schulze et al., 2020). These mechanisms need not be mutually exclusive; different plant species can lie at various points along a continual spectrum of response. Failure to acclimate to stress due to insufficient avoidance or tolerance mechanisms can result in a number of deleterious events, which in the extreme can lead to death. Plant tolerance of stressful conditions can be due to physiological mechanisms of resistance or resilience. Resistance in this context means maintenance of growth, development, and productivity under stressful conditions, while resilience means that plants show reduced rates of growth or productivity under stressful conditions, with some degree of increase either to the pre-disturbance level or to a new stable condition, after the stress condition is past. Plant resilience can vary both in how quickly the plant will be able to return to the pre-disturbance condition (Holling, 1973; Lloret et al., 2011; Eilmann and Rigling, 2012) or in the difference between the pre-disturbance condition and the new stable state (Ingrisch and Bahn, 2018). In the particular case of forest species, studies are often motivated by how to enhance productivity and define the level of tolerance or resistance by the maintenance of stem growth and wood production (e.g., Orwig and Abrams, 1997; Eilmann et al., 2010). Nevertheless, the levels of stress-response and survival rates depend to a great extent on the initial plant condition (e.g., type of species, age), the plant history (e.g., priming, or acclimation to previous stressful events), and the level and extension of the disruption.

This is particularly interesting in the case of cultivated forest tree species for which genetic improvement programs exist and provide a significant fraction of planting stock for reforestation. In these species, breeders have typically focused on selection of trees with improved productivity and quality traits under climate conditions that were expected to vary little over time. Unfortunately, climate change is associated with increases in both the variability and the average levels of temperature and water availability, leading to extreme variability of weather events recently called “weather whiplash.” This phenomenon has been highlighted as a major issue that will affect the way plants and animals will mitigate and adapt to future climate conditions (Swain et al., 2018; Casson et al., 2019). These changes have the potential to degrade the productivity of cultivated forest species, so tree breeders will need to incorporate some measures of tolerance or resistance to these disturbances into their breeding and selection programs as soon as possible in order to minimize negative impacts on productivity of forest plantations in the latter half of this century.

Managed agricultural and forest ecosystems can be modified both through changes in management practices and by choice of resilient and well-adapted planting stock, but stress response plasticity and the genetic differences that underline tolerance and resistance are still largely unknown in conifers. The combination of new phenotyping technologies and genomic tools has allowed major advances in understanding of the genetic basis of abiotic stress response in Populus species (e.g., Georgii et al., 2019), particularly Populus trichocarpa (e.g., Weighill et al., 2019a,b). These new technologies in the areas of engineering and genomics are beginning to be applied to phenotype-genotype studies in conifers as well (D’Odorico et al., 2020; Depardieu et al., 2020). Continued advances in acquisition and analysis of data using sensing technologies such as hyperspectral imaging must be combined with advances in genomic tools and resources to better understand the role of genetic and epigenetic mechanisms in conifer responses to drought and cold stress, and find new paths forward in forest tree breeding.

Loblolly pine (*Pinus taeda* L.) is the most-planted forest tree species in North America (McKeand et al., 2021), and will be the species on which our review is focused. We begin by introducing the extensive geographic variation observed for loblolly pine and the importance of selecting an appropriate seed source to ensure good survival and optimal productivity. We then introduce a general timeline of loblolly pine seed production and plantation establishment, and highlight the stages most vulnerable to extremes of temperature and drought. This is followed by a review of the physiological changes that have been described as adaptation strategies adopted by plants to cope with drought and cold stress. This topic offers a perfect transition into the molecular level and a relatively new area of research that studies the cross-talk between stress responses and the potential to use this information to identify trees with increased adaptation and resilience. We conclude with a discussion of the potential of high-throughput phenotype data collection and genomic analyses to enable new approaches to improving resistance or resilience of forest trees to drought and cold stress. Considerable progress has been made in this area with angiosperm forest trees (Tuskan et al., 2018), but much less is known about conifers due to their much larger and more complex genomes; our focus will be on conifers in general and loblolly pine in particular.

## Loblolly Pine Breeding and Production

### Natural Range and Geographic Variation

**FIGURE 1:**
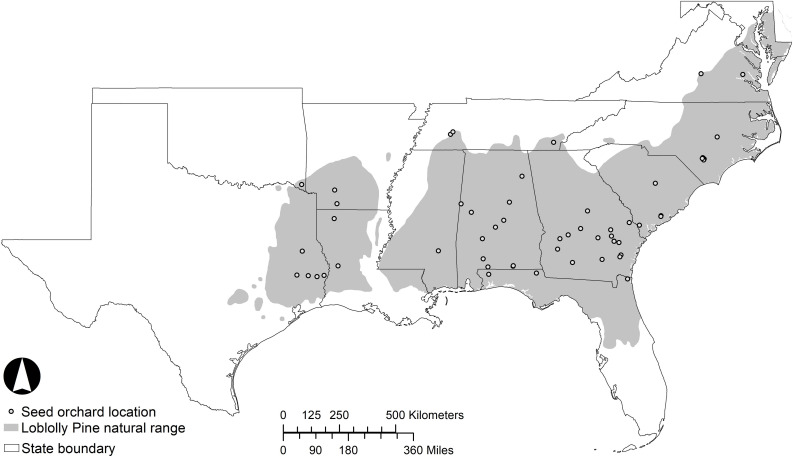
The inset shows the southeastern region of the United States, and the shaded area of the figure shows the natural range of loblolly pine within the region. Dots indicate the approximate locations of active loblolly pine seed orchards.

Loblolly pine is the most widely planted timber species in the southeastern United States (Schultz, 1997). The greatest productivity of planted pine is achieved through the practice of intensive silviculture, where superior genotypes are selected to match the site and subsequent silvicultural treatments are directed at alleviating resource limitations (Allen et al., 2005; McKeand et al., 2006; Fox et al., 2007). Loblolly pine naturally inhabits a diverse range of environments, extending from Delaware to central Florida and west to eastern Texas (Schultz, 1997). The distribution is mostly continuous (Figure 1), with a major discontinuity at the Mississippi River Valley and a few small disjunct populations in Texas collectively known as the “lost pines.” In general, the natural range of loblolly pine is limited to the north by low temperature and to the west by low precipitation (Schmidtling, 2001). Given the wide geographic range with differing climates and photoperiods, this species has extensive geographic variation.

Research shows that the selection of an appropriate seed source of loblolly pine is crucial to ensuring good survival and optimal productivity. A pioneering study involving the establishment of a loblolly pine provenance test in 1927 in eastern Louisiana (Wakeley, 1944; Wakeley and Bercaw, 1965) highlighted the existence of genetic variation across a wide geographical distribution. In the study, the trees grown from the local seed source of Livingston Parish, LA produced approximately twice the volume of wood per hectare as those grown from seeds sourced from Arkansas, Georgia, and Texas. The general conclusion was drawn that local seed sources are best, although the study was planted in only a single location. A number of studies have expanded on this work to characterize patterns of geographic variation in the southern pines, including the Southwide Southern Pine Seed Source Study (SSPSSS) initiated in 1951 (Wells and Wakeley, 1966; Wells, 1983). The results of the SSPSSS indicated that while the Livingston Parish provenance was indeed superior on many sites, it is not always true that local seed sources are superior for economically important traits compared to more distant sources. For example, seed sources from warmer regions tended to grow faster than local sources, providing the difference in the average minimum winter temperature between the source and planting location was not too great (Schmidtling, 2001).

Average minimum winter temperature is commonly used by horticulturalists to guide seed transfers (Pike et al., 2020), and forms the basis of the USDA Plant Hardiness Zones (USDA Plant Hardiness Zone Map, 2012). These zones are based on increments of 5° to 10° F (2.8–5.4 C) for the convenience of US gardeners. Analysis of a variety of provenance and seed source studies led to the conclusion that loblolly pine seed sources can be planted in an area having an average minimum winter temperature up to 5° F (2.8°C) below that found at the seed source, and will typically result in an increase in productivity relative to seeds local to the planting area with minimal risk of cold damage (Schmidtling, 2001; Lambeth et al., 2005). However, these authors caution that the transfer of seed northward or inland to a planting site with winter minimum temperature colder by more than 10 °F (5.4°C) can result in an elevated risk of cold damage. With the prospect of warmer temperatures as a result of climate change, the economic advantage of moving loblolly pine from seed sources of warmer regions to colder planting sites approaching a temperature differential of 10 °F (5.4°C) or more, will need to be balanced with the risk of cold damage during extreme freeze events.

Important observations regarding the movement of seed longitudinally were also brought to light by the SSPSSS. Possibly the most important observation was that seed sources from west of the Mississippi River were more drought tolerant and disease resistant to fusiform rust disease (caused by the fungus *Cronartium quercuum* f. sp. *fusiforme*) compared to eastern sources but that the western sources generally grew slower (Wells, 1985; Schmidtling, 2001). The presence of this variation is likely rooted in the Pleistocene geologic era, as it is postulated that loblolly pine survived the Pleistocene glaciation of North America in two separate refugia, one in southeast Texas and or northeast Mexico, and the other in south Florida and or Caribbean (Wells et al., 1991; Schmidtling et al., 1999; Schmidtling, 2003). The appreciation of these patterns of geographic variation was an important first step in the genetic improvement of loblolly pine.

Tree improvement programs in the southern United States have structured the genetic resource of loblolly pine into breeding populations that broadly represent the different geographic regions of the species. Tree breeders have primarily focused on improving volume growth, tree form, disease resistance, and wood quality (Zobel and Talbert, 1984). In the 1950s, several large tree improvement programs were initiated at land grant universities and at the USDA Forest Service in order to meet the growing needs of plantation forestry in the southern United States (Zobel and Sprague, 1993). Improved seed from first-generation seed orchards became available in the 1960s and early 1970s, and produced 7–12% more volume per hectare at harvest than trees grown from wild seed (Li et al., 1999). Over time, tree improvement programs in the region have transitioned to the second, third, and fourth cycles of improved material (McKeand and Bridgwater, 1998). Aspinwall et al. (2012) estimated the realized gain in productivity from planting genetically-improved loblolly pine was 17% for the period from 1968 to 2007. Currently, approximately 80% of the more than 12 million planted hectares in the southern United States are loblolly pine, with upwards of 700 million seedlings deployed annually (McKeand et al., 2015) from Virginia in the north, south to Florida and west to Texas. Numerous cases of operational plantations have demonstrated large realized genetic gains from using non-local seed sources, delivering benefits that include increased growth, reduction in fusiform rust disease, improved cold or drought tolerance, and better stem form and wood quality (Lambeth et al., 2005). However, the establishment of non-local provenances does come with risks associated with biotic and abiotic stress, particularly cold or drought damage. Hence, thorough testing of improved genetic material remains important to ensure the successful establishment and desired productivity of planted forests.

### Climate Change and Its Impact on the Production and Deployment of Loblolly Pine

**FIGURE 2:**
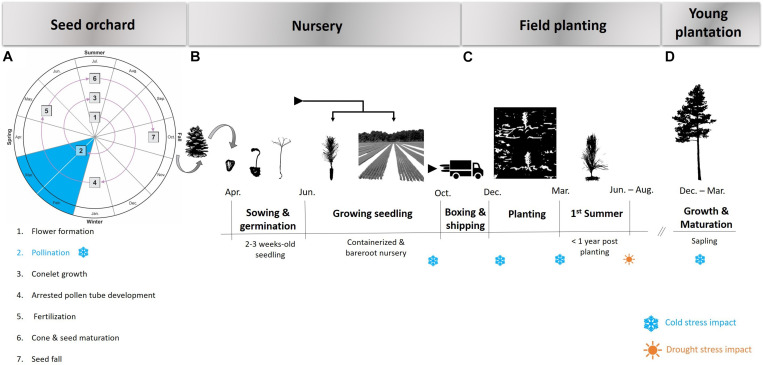
Process flow of loblolly pine seed production (Adapted from Bramlett et al., 1978) **(A)**, nursery raising **(B)**, field planting **(C)**, and early growth **(D)**. The possible occurrence of cold and drought events resulting in an elevated risk of stress is indicated in blue and orange respectively.

The typical cycle of planted loblolly pine forests commences in the seed orchards where improved seed is produced and made available to nurseries that grow seedlings for plantation establishment. During each of these stages there is the potential for cold and/or drought events (Figure 2). The ability of planting stock to withstand extremes of temperature and water availability at different times of the year or at different stages of the life cycle is important to quantify and manage in both the seed orchard and deployment environment.

Loblolly pine seed orchards are established throughout much of the southeastern United States with a high concentration in the southern regions of the states of Georgia and Alabama (Figure 1), but all within the natural range of loblolly pine. Seed orchard managers often select sites in a less harsh environment than the seed source origin with respect to minimum winter temperature, with the major concern being the detrimental effects of extreme cold events on strobilus development and survival. Abundant survival of female strobili is a prerequisite for a plentiful cone crop. In loblolly pine, strobilus primordia develop rapidly when spring temperatures begin to rise in mid-to-late February and early March (Schultz, 1997). Pollination typically occurs from February to April, with the window of pollen shed and female receptivity shifting from south to north along the species distribution as warmer temperatures move northward. Female strobili are most susceptible to spring freeze events during the time of female receptivity and post-pollination pollen tube growth (Figure 2A). Fertilization occurs over a year later, once the pollen tube reaches the archegonium in the summer of the year following pollination. The effect of temperature and other external variables on pollen- development, kinetics and dynamics is highly unexplored in loblolly pine. Thus, improvement of pollen-collection practices and pollen-viability tests could be part of future studies. Mature cones are harvested in October, approximately 4 months after fertilization and eighteen months after pollination.

Knowledge of the molecular mechanisms controlling reproductive processes in conifers is limited, so the potential for improving the resilience or resistance of loblolly pine seed production is unclear. Some genes related to male strobilus and pollen development have been characterized, including MADS-box genes (Sundström and Engström, 2002; Katahata et al., 2014), FT (FLOWERING LOCUS T)-like genes (Klintenäs et al., 2012), gibberellin metabolism genes (Niu et al., 2014), and putative LEAFY homologs (Dornelas and Rodriguez, 2005; Shiokawa et al., 2008). A few reports of transcriptome analyses of conifer reproductive tissues have been reported (Niu et al., 2016; Ma et al., 2020), and as the quality of conifer genome assemblies and annotation is improved, these data may contribute to understanding of molecular mechanisms that will enable better management of abiotic stress effects in conifer seed production.

Seed harvested from pine orchards are typically sown in forest tree nurseries, and the seedlings are cultivated as field-grown bareroot stock or seedlings grown in containers. Irrigation systems ensure that water stress is mitigated in the seedling nursery setting. However, the degree of control over factors affecting freeze injury of open grown seedlings in the nursery is more challenging. At a macro scale, nurseries should be located in a cold hardiness zone that is appropriate for the level of cold adaptability of the seed source. For example, an increased likelihood of freeze injury may occur if seed sources of southern pines from hardiness Zone 8 are sown in nurseries located in Zones 6 or 7a (South, 2007). Seeds are typically sown after the risk of frost is past in the location of each nursery, so the first significant risk of cold exposure comes in the fall when the seedlings are 5- to 6 months old (Figure 2B). The topography of the nursery site also impacts the level of exposure of seedlings to freezing temperatures. Obstruction of cold air drainage by land, buildings, or vegetation can result in the ponding of cold air and greater risk of freeze damage (Colombo et al., 2001). The gradual cold hardening process is triggered in the fall by shortening photoperiods and lowered temperatures (Weiser, 1970; Aronsson, 1975; Greer and Warrington, 1982). Conversely, lengthening photoperiods and, in particular, increased temperatures provide cues for rapid dehardening (Greer and Stanley, 1985; Leinonen et al., 1997). The greatest risk of cold damage is caused by unseasonal frosts in the fall while the plants are still hardening, and in the spring when plants are dehardening (Bannister and Neuner, 2001). A freeze event preceded by unseasonably warm temperatures is likely to increase the risk of freeze injury. South (2006) provided a summary of historical freeze events that occurred in the southeastern United States and their impact on pine seedlings grown in nurseries across the region. The findings show a trend that unusually high minimum temperatures for several days prior to a hard freeze increases the likelihood of seedling cold damage, compared to freeze events preceded by seasonally typical (lower) temperatures. Consequently, a future of warming temperatures and increased climate variability may adversely affect levels of cold hardiness and render conifers more susceptible to injury brought about by freeze events (Bansal et al., 2015).

For planted forests, it is during the first year following field establishment that the crop is most vulnerable to cold and drought stress (Figure 2C). At this stage, plants are small and have relatively undeveloped root structures with limited access to water and nutrients, which exacerbates their susceptibility to stress factors. Prolonged dry periods during the summer months following planting are particularly challenging in excessively drained and shallow soils that accentuate the effects of drought. Numerous climate models predict future drier conditions in much of North America, including the southeastern United States (Dai, 2013; Cook et al., 2014). For loblolly pine, provenances in the western portion of the natural range have evolved in more xeric climates and have likely responded to natural selection for drought resistance (Schmidtling, 2001, 2003). The results of long-term field trials established on relatively dry sites in Texas showed that local Texas seed sources were on average more productive than non-local seed sources (Long, 1980). Studies of tree mortality after a severe drought in Texas in 2011 reported that mature loblolly pine trees showed lower mortality than other pine or angiosperm tree species in the region (Klockow et al., 2018, 2020). Two long-term trials in southern Arkansas, where water availability is less limiting, showed that loblolly seed sources from east of the Mississippi provided significantly more volume growth compared to local seed sources (Wells and Lambeth, 1983). An example of the successful movement of seed sources from east to west for commercial operations involved the planting of North Carolina loblolly pine in Arkansas and Oklahoma (Lambeth et al., 1984). In those operations, the risk of drought damage was reduced by planting the non-local planting stock on sites deemed to have an appropriate soil profile to ensure water availability. The successful transfer of faster growing seed sources from east to west on suitable soils will likely continue, although the potential for decreased rainfall due to climate change suggests this practice should be pursued with caution.

## Physiological Responses to Water Deficit and Cold Stress

Among the abiotic stresses that affect plants, suboptimal temperature and water deficit are particularly critical for determining plant survival. The two major mechanistic hypotheses to account for drought-induced tree death are hydraulic failure and carbon starvation. During hydraulic failure tree death is largely explained as a result of dysfunctional water transport caused by xylem embolism, whereas during carbon starvation death is caused by shortages of carbohydrate reserves resulting from a decline in photosynthesis (McDowell, 2011; Sevanto et al., 2014). The precise manner in which these carbon and water relations play out during extreme drought, especially under field conditions, remains unclear. As a result, new studies have reframed our understanding of plant hydraulics by introducing the interactions between a tree’s physiological strategies within a local environment (Sperry and Love, 2015; Feng et al., 2017), their phenotypic characteristics (Couvreur et al., 2018), phenotypic plasticity, and the dynamic spatial interactions of these variables (Manzoni et al., 2014). The introduction of multidimensional models to understand tree water management has opened opportunities to obtain a more accurate understanding of how trees in natural and managed forests respond to restricted water availability and the variation of this response in a future climate change. Among these topics, researchers have studied more carefully the interactions between carbon metabolism and hydraulics (McDowell et al., 2008; McDowell, 2011), the negative legacy of drought on tree stem growth (Anderegg et al., 2015), the implications of the use of storage water to reduce fluctuations in xylem tension (Skelton, 2019), and the extent to which xylem refilling may contribute to the reversibility of loss in xylem hydraulic conductance over longer timescales (McCulloh and Meinzer, 2015; Trifilò et al., 2015). Two major physiological strategies plants use to mitigate the stress of water deficit are avoidance and resistance. Avoidance, in the context of plant response to water deficit, could be defined as the ability to maintain fundamental normal physiological processes under mild or moderate drought stress conditions by reducing water loss (e.g., rapid stomatal closure), enhancing water uptake ability (e.g., changes in root morphology), and modifying vegetative and reproductive growth. On the other hand, drought resistance refers to the ability to maintain physiological activities under severe drought conditions by increasing osmoregulatory molecules in the cells, and adjusting enzymatic pathways to reduce the accumulation of hazardous substances (Fang and Xiong, 2015).

### Iso/Anisohydric: A Complex Hydraulic Trait

Trees, like other terrestrial plants, transpire most of the water extracted from the soil in exchange for CO_2_ in the atmosphere to convert solar energy, CO_2_, and water into organic matter and oxygen. A sophisticated hydraulic arrangement allows the long-distance transportation of water that relies on the maintenance of water potentials along the root-trunk-branch-leaf continuum and the interaction soil-plant-atmosphere (Feng et al., 2017). The terms isohydric and anisohydric refer to water management strategies that plants use to maintain optimal water capacity by the control of stomatal aperture. This helps plants to maintain a basic tradeoff between carbon gain and water loss and has been linked to plant vulnerability to hydraulic failure (e.g., embolism) or carbon starvation. The terms have been re-evaluated over the last two decades as understanding plant water-relations has grown in connection with studies of the mechanisms that triggered massive numbers of tree deaths in different ecosystems globally (Allen, 2009; O’Brien et al., 2017). The anisohydric/isohydric dichotomy has proven to be insufficient to explain the multifunctionality of hydraulic traits that trees use under stress conditions and cannot be used as a unique indicator of drought-induced vulnerability or mortality between or within species (Klein, 2014; Martinez-Vilalta and Garcia-Forner, 2017). For example, in conifers, relatively isohydric species like *Pinus radiata* (Mitchell et al., 2017) rely on high levels of the hormone abscisic acid (ABA) to maintain stomata closed during sustained water stress. In loblolly pine and *Pinus edulis* (Plaut et al., 2012), other relatively isohydric species, drought-induced mortality has been hypothesized to be the product of long-term stomatal closure rather than hydraulic failure from cavitation itself (McDowell et al., 2008), while other conifers have xylem tissues with extreme resistance to embolism allowing leaves to dehydrate (Brodribb et al., 2014). Furthermore, researchers have found that trees can also exhibit differences of those traits in different organs (Domec et al., 2009; McCulloh and Meinzer, 2015), despite the observation that forest ecosystems around the globe have a tendency to maintain similar margins for water potentials (Sperry and Love, 2015) and hydraulic conductivity and capacitance (McCulloh et al., 2013) as mechanisms to avoid stem dieback and death. However, as Rodríguez-Gamir et al. (2016) state, there is inconsistent information on the contribution of different plant organs to the total hydraulic conductance and respiration.

Phenotypic plasticity in water−use traits and evidence of the strong impact of environmental conditions on drought-stress responses have been documented for loblolly pine. In one of these studies, genetically related and even-aged loblolly pine trees were exposed to different water constraints imposed by different soils. The trees that grew in soils of lower water availability (i.e., sand), had longer root systems and were more prone to xylem cavitation (Hacke et al., 2000). Another group investigated the effects of changes in CO_2_ and nitrogen on water transport traits, long distance water transport, and drought tolerance in loblolly pine trees in a plantation on low clay loam. The study revealed that loblolly pine trees required a structural change of the hydraulic pathway to produce stomatal closure under elevated CO_2_ and nitrogen, attributing these changes, at least partially, to the development of conducting tissue with different hydraulic characteristics. Interestingly, they also demonstrated that under certain circumstances trees that grew under elevated CO_2_ conditions showed less reduction in stomatal conductance than trees that grew under normal CO_2_, similar to the response observed in broadleaf trees. This could imply that measurable changes might be correlated to the time of exposure and acclimation to elevated CO_2_ by the alteration of the anatomy of loblolly pine needles (Domec et al., 2009, 2016). Other studies in 2-year-old loblolly pine saplings were focused on the correlation between temperature and the regulation of stomatal adjustment. Elevated CO_2_ caused a decline in stomatal conductance, as it was observed before by Domec et al. (2009), whereas an increase in leaf temperature had the opposite effect. Nevertheless, the increase in the level of CO_2_ did not fully mitigate the increased stomatal opening when both temperature and CO_2_ concentration increased. Surprisingly, when comparing the response of loblolly pine with poplar, a species that uses a completely different management strategy for water loss, both species increased stomatal conductance under elevated temperatures, suggesting that the regulation of stomatal activity did not depend exclusively on the rate of transpiration. The inability of loblolly pine to maintain strict control over water loss by transpiration when temperatures increased may have a negative impact on survival and productivity under a hotter future climate but more such studies must be performed in the field to obtain more accurate predictions (Carnicer et al., 2013; Urban et al., 2017).

### Embolism and the Complex Dynamic for Hydraulic Capacity Recovery

Cavitation is a phenomenon that occurs in xylem of vascular plants when the pressure of the water phase falls below its vapor pressure and changes from liquid to gas creating a bubble that fills the vessels or the tracheids. The blocking of a xylem vessel or tracheid by a bubble is called embolism. There are two known environmental causes of xylem cavitation: water stress (drought) and freeze-thaw cycles (frost drought) (Tyree and Sperry, 1989). In the case of water stress, the evidence indicates that critical low xylem pressures aspirate bubbles into the functional xylem conduits through pit membranes communicating with previously embolized conduits (Jarbeau et al., 1995) while in freezing, the air bubbles can form *in situ* because gases are insoluble in ice (Ewers, 1985). Despite the unanimous agreement in the relationship between drought-induced mortality and resistance to xylem embolism (Adams et al., 2017; Choat et al., 2018), a debate has arisen regarding the ability of some techniques to measure and discern between the capacity of a plant to resist embolism (Brodribb et al., 2010; Delzon and Cochard, 2014; Choat et al., 2016), and the ability to recover by use of other strategies. A recent publication exemplified this controversy by rebutting a well-known theory about the high vulnerability to embolism and embolism repair mechanisms in laurel (*Lauris nobilis* L.) (Salleo et al., 1996; Tyree et al., 1999; Trifilò et al., 2014, 2015). The authors used a direct, non-invasive method and concluded that this species is not only highly resistant to xylem embolism but that in absence of extreme drought events, it will maintain a positive hydraulic safety margin, and daily cycles of embolism formation and refilling are unlikely to occur (Knipfer et al., 2015, 2016; Lamarque et al., 2018). This common disagreement lies, at least partially, in our limited ability to observe *in vivo* the functional status of xylem conduits (Klein et al., 2018). The idea that trees can establish the positive pressure required to remove emboli in transpiring tissues many meters above the soil surface has been deemed unlikely by some authors, making refilling under sustained tension within the current theoretical framework thermodynamically untenable (Zwieniecki and Holbrook, 2009; Brodersen and McElrone, 2013).

Evergreen conifers suffer frost drought when the ice in soil, roots and frozen trunk parts prevent water uptake for months, while needles can reach substantially higher temperatures than the air during sunny winter days causing relevant water losses (Mayr et al., 2003, 2006). Recent publications have provided more evidence of these mechanisms in evergreen conifers. Mayr et al. (2019) analyzed the loss of hydraulic conductivity in a 10-year dataset, and correlated winter embolism to climate parameters. They demonstrated that *Picea abies* is able to survive winter embolism based on periodic xylem refilling during spring. On the other hand, Hammond et al. (2019) were interested in identification of a lethal threshold for hydraulic failure when portions of the xylem were still functional but the conductivity was reduced below a threshold for sufficient survival even when water became available. As a result, they found that the point of no return for 2-year-old loblolly pine saplings was a threshold at 80% loss of hydraulic conductivity, although certain trees were able to survive even when more than 90% of their xylem conductivity were lost. This threshold was much higher than what has been reported for other conifers (Brodribb and Cochard, 2009). The authors discussed that the possible cause for this extreme survival-trait could be attributed to a non-gradual re-watering during drought relief because in the field, rewatering usually never happened at once and in a continuous way. They also concluded that as long as there was supply of water to the vascular cambium, and xylem tension was able to relax before complete hydraulic failure, there was a chance for survival. Finally, the generated function for calculating mortality could be directly input to vegetation models, although the authors suggested more studies to standardize the analysis across different developmental stages and species. It is important to notice that vulnerability to cavitation does not determine drought tolerance itself. The establishment of the critical threshold and the time to reach it, are the results of the interaction of a number of associated physiological, morphological and environmental factors and therefore, further experimental studies are required to confirm the processes involved in drought-recovery (Choat et al., 2018).

### The Paradox: Water Use Efficiency and Fertilization

For loblolly pine plantations, productivity has been the result of years of intensive management and silviculture advancements that have enabled a higher profitably for a variety of uses in very short rotations (8–12 years) (Coyle et al., 2016; D’Amato et al., 2017). The strategy of the plantations is to increase leaf area or water availability, while stem growth is often predicted by leaf area index. In the southeastern United States the major limitation to loblolly pine production is site fertility, specifically related to available nitrogen and phosphorus, even on excessively drained soils (Fox et al., 2007). Mid-rotation fertilization has been an increasingly common practice to provide the nutrients to redirect growth from roots to leaves and stems (Albaugh et al., 2004). Recent studies in loblolly pine have focused on unraveling how fertilization and water availability affect growth, and water use efficiency to predict tree and stand growth but have found very contrasting results. Samuelson et al. (2014) reported no effects of fertilization or throughfall reduction on growth efficiency of a 7-year-old loblolly pine plantation in Georgia. In contrast, a study conducted in a well-drained loblolly pine plantation in Virginia, when the trees were in their 8th and 9th growing seasons, concluded that during the growing season, the combination of fertilization and a reduction of throughfall caused the most consistent decrease in canopy-averaged stomatal conductance and canopy transpiration but apparently did not limit photosynthesis, causing a significant increase in stem volume (Ward et al., 2015). Interestingly, according to these authors, the leaf area index (LAI) did not increase with fertilization and they suggested that this could be related to the stand having not reached the point at which its nutrient demand exceeded the current supply. They also suggested that the largest changes might occur in the hydraulic system structure, specifically in the root system. This was in agreement with Ewers et al. (2000) who concluded that fertilized stands reduced the safety margins to avoid hydraulic failure and the reduction in the root system made loblolly pine more susceptible to drought-induced mortality. Maggard et al. (2017) found that fertilization increased LAI in the two first years of their study but it was not significant in the third one, attributing the result to a weakening effect of the one-time fertilizer treatment 3 years after application. They also found a negative effect of throughfall reduction on LAI, in contrast with no effect on LAI found after ∼30% reduction on a 7-year-old loblolly pine plantation in Georgia (Samuelson et al., 2014), and over the 2 years for a 9-year-old loblolly pine plantation in Virginia (Ward et al., 2015). Despite these findings supporting negative impacts in loblolly pine plantations due to water scarcity, the current models seem to be affected by local variables and stand traits that should be evaluated before inferring direct effects on productivity.

### Theory Interplay: Carbon Starvation and Hydraulic Failure

Plant carbon starvation is the physiological result of an imbalance that occurs when carbon supply from photosynthesis, non-structural carbohydrates (carbon storage compounds) and metabolites from autophagy are not enough to maintain the carbon demands for respiration, growth and defense (McDowell, 2011). Maintaining a positive carbon balance during drought conditions is often considered a major challenge for trees (Galiano et al., 2011; Mitchell et al., 2013) but even during prolonged drought there is more than one way for trees to maintain a positive carbon balance (Klein, 2014; McDowell et al., 2016). To maintain this balance, trees must control stomatal aperture. Although a reduction in the stomatal conductance has been proven to be insufficient to explain mortality directly, theory and evidence point that a reduction in hydraulic function and photosynthesis caused by a decline in stomatal conductance, are primary drivers of death (McDowell, 2011; McDowell et al., 2013). Results from different studies provided clear evidence of partial and complete plant mortality associated with hydraulic failure in both isohydric and anisohydric species, however, no tests of hydraulic failure have excluded carbon starvation or other processes as mechanisms involved in mortality (McDowell, 2011). Furthermore, woody plants exhibit a continuum of hydraulic strategies, rather than a clear distinction between two contrasting alternatives (Sevanto et al., 2014), as well as intraspecific variability in water-use strategies to either resist or recover from different levels of drought stress (Poyatos et al., 2013; Hentschel et al., 2014; Klein, 2014; Garcia-Forner et al., 2017; Petrucco et al., 2017). A large number of recent experiments have proven that hydraulic failure and carbon starvation are interconnected processes (Savi et al., 2016; Yoshimura et al., 2016; Petrucco et al., 2017; Tomasella et al., 2017) and attempts to underline their interplay depends on the intensity and the duration of the drought event (McDowell et al., 2016). Drought duration is important because stored carbohydrates may act temporarily as a buffer that could supply carbon to continue with basic physiological processes even under severe drought events, but extended droughts can increase pest vulnerability (McDowell et al., 2008, 2016) and this could be an additional variable that could affect tree mortality.

Allocation of carbon is a mechanism that plants used to adapt to changes in the environment (Chaves et al., 2002). The distribution of carbon among the plant under stress conditions is correlated to the production of recent photosynthates and the carbon stores and their accessibility. Recently, new research is providing the first steps toward quantifying response thresholds for carbon allocation in different species including evergreen trees under stress conditions (Blessing et al., 2015; Eziz et al., 2017). Bradley and Will (2017) explored biomass distribution and its correlation with transpiration rates for shortleaf pine (*Pinus echinata*), loblolly pine, and their hybrids under limited water conditions. Drought tolerance is linked to survival in the first years of planting and therefore to long-term productivity, and “shortleaf pine is presumed to be more drought tolerant than loblolly pine” (Bradley and Will, 2017), so the authors wanted to determine drought hardiness in the hybrid while unraveling biomass partitioning. The authors found that shortleaf and the hybrid pines had a greater allocation of carbon in the roots, and speculated that this could lead to better performance by shortleaf and hybrid pines under drought conditions. It is interesting to note that an analysis of tree mortality across 5 years after a severe drought in east Texas in 2011 reported that loblolly pine showed significantly lower mortality than shortleaf pine in years three, four and five (Klockow et al., 2018). Despite the large list of publications on this topic, the minimum carbohydrate threshold that determines plant survival, as well as the responses to the co-occurrence of carbon starvation and hydraulic failure processes is unknown.

## Phenotypic Plasticity and Signaling Crosstalk Under Stress

Phenotypic plasticity is an incredible evolutionary tool that confers any organism the ability to adapt to and cope with changes in its environment. Plastic responses depend on changes in gene expression and protein function and guarantee the maintenance of metabolic homeostasis, foraging for resources, and defense (Aphalo et al., 1999). Therefore, they are expressed over a variety of time scales, from the molecular level during short-term acclimation, to changes at the morphophysiological level that may take several years to develop. For generations, traditional tree breeders have been able to improve traits that have an economic and environmental impact based on the selection of phenotypes since they reflect the interactions between the genome and a complex network of responses to the micro-and mega- environments. Nevertheless, fast and unpredictable changes in climate to which some species may be unable to rapidly adapt, and the physiological nature of woody species, are slowing the development of effective tools for tree improvement. Genes that are important in establishing stress responses are being identified thanks to the rapid growth of genomics. In recent years, reduced sequencing cost and high throughput systems have increased the available transcriptome datasets and the number of scientists who attempt to sequence and assemble complex tree genomes. Reference genome sequence assemblies have been published for ten species of gymnosperms: *Picea abies* (Norway spruce) (Nystedt et al., 2013), *Picea glauca* (white spruce) (Birol et al., 2013; Warren et al., 2015), *Pinus taeda* (loblolly pine) (Neale et al., 2014; Wegrzyn et al., 2014; Zimin et al., 2014), *Pinus lambertiana* (sugar pine) (Stevens et al., 2016), *Ginkgo biloba* (ginkgo) (Guan et al., 2016), *Pseudotsuga menziesii* (Douglas fir) (Neale et al., 2017), *Gnetum montanum* (Wan et al., 2018), *Larix sibirica* (Siberian larch) (Kuzmin et al., 2019), *Abies alba* (European silver fir) (Mosca et al., 2019), and *Sequoiadendron giganteum* (giant sequoia) (Scott et al., 2020). The long term goal of plant genomic studies is to accelerate our understanding of the networks involved in both the normal- and the stress- functioning of the organisms, thus accelerating the breeding process. In this genomic era, new methods to accelerate breeding, improve resistance, and increase genetic gain have promised to be the future for agriculture. This includes whole-genome based selection and even customized genotypes by the use of editing techniques like CRISPR/Cas9. In order to achieve this goal in conifers, scientists need to overcome two major limitations. First, the sequencing of conifer megagenomes requires large investments that most public and private investors are not willing to pay. This is, to some extent, related to the second limitation, in that once the DNA sequence data are in hand, the assemblies are often highly fragmented. Many of the assemblies listed above contain over a million scaffolds (Gonzalez-Ibeas et al., 2016; Mosca et al., 2019). A large investment of time and computational resources will be required to improve these fragmented assemblies. The one exception to this is the genome of *Sequoiadendron giganteum* or giant redwood; Scott et al., 2020 reported assembly of eleven chromosome-scale contiguous sequences totaling 8.125 Gb (the expected haploid genome size). The combination of sequencing technologies and assembly methods used for this project may be the guide for future efforts to improve the quality of other conifer genome assemblies.

According to Knight and Knight (2001), cross-talk is defined as the convergent result from different stressors while specificity is referred to as any part of a signaling pathway that enables the distinction between two or more possible outcomes. During cross-talk different pathways could achieve the same response, or interact with other pathways, each affecting the other’s outcome. The perception and response to stress signals occur via secondary messengers including cyclic nucleotides, lipid molecules, reactive oxygen species, H_2_O_2_, nitric oxide and Ca^2+^(Zhou et al., 2019). Since they also play important roles in the maintenance of homeostasis, changes in their production, balance, use, and elimination are commonly related to stress signaling. Then, the transcription factors and their co-regulators are the key nuclear effector proteins responsible for translating cellular inputs created by the secondary messengers to target and control dosage of gene expression. This can be achieved by post-transcriptional modifications of the transcription factors and their co-regulators. Finally, the activation or suppression of stress-responsive genes can change the levels of proteins that have either metabolic or regulatory roles, such as those involved in detoxification, osmolyte biosynthesis, proteolysis of cellular substrates, water channels, ion transporters, and heat shock proteins (Joshi et al., 2016). Only recently have researchers started to pay more attention to the molecular mechanisms that occur when the stressors appear almost simultaneously, decreasing the time that plants require for adjustment and recovery (Hussain et al., 2018). Simple strategies for improving the performance of trees in water-limited environments do not exist, mainly because the responses are the results of multiple tolerance and avoidance mechanisms (Polle et al., 2019). Among the reviewed literature [Polle et al., 2019; see Li X. et al. (2019); Estravis-Barcala et al., 2020], authors agreed that the identification of the genes expressed or inactivated during stressful events, that are also common features of different stress-response pathways, could play a key role in enhancing tree tolerance to multiple harsh environments. However, the inference and extrapolation of information from genomic studies in model plants to the analysis of molecular events in trees must be done cautiously, not only because of the multiple variables during the performance of the experiments but also because of the fragmented nature of the current tree genomes. Furthermore, a unanimous conclusion was found in all studies, the imperative necessity to characterize stress responses in large-scale experimental field studies in different environments and among different developmental stages.

## Post-Transcriptional Regulation and Stress Memory

### Alternative Splicing and Alternative Polyadenylation

Transcribed RNA is modified by the addition of a 5’ cap structure, transformation into a mature messenger RNA (mRNA) by splicing, and the addition of a 3’ polyA tail by polyadenylation. Plant studies have shown that alternative splicing (AS) and alternative polyadenylation (APA) are important mechanisms involved in the regulation of the proteome, but there is only a limited knowledge of the interaction between these mechanisms and their interplay during transcriptional and post-transcriptional regulation in plants. During AS, multiple messenger RNA (mRNA) isoforms are produced from a single gene enhancing the functional diversity of the proteome during development (Szakonyi and Duque, 2018) and under stress conditions (Marquez et al., 2012; Chamala et al., 2015; Thatcher et al., 2016; Mei et al., 2017). APA, confers a gene the capacity to generate transcripts with multiple polyadenylation sites and differential usage of these sites can lead to the formation of distinct mRNA isoforms (Edwalds-Gilbert et al., 1997; Barabino and Keller, 1999). Natural genetic and phenotypic variations that occur in crop plants constitute the main resources for modern breeding strategies. In several crops, AS has shown to be an efficient tool to improve stress-resistance and to have a direct effect on yield and growth (Panahi et al., 2014; Mei et al., 2017; Wei et al., 2017). Due to cost and technological limitations, the number of studies that have been successful at producing high-quality genome references is still smaller than the constantly growing number of transcriptome studies. High throughput sequencing can be applied in species with and without reference genomes but detection of AS and APA events depend on the accurate prediction of transcript sequences, hence depending on the ability to reconstruct full-length isoforms and high-quality transcriptome annotations. Unfortunately, for many plant species including gymnosperms, the knowledge of which parts of genomes constitute genes and their isoforms remains unclear. This has been a notorious bottleneck in the study of AS and APA in non-model plants, particularly woody species with complex genomes. Chen et al. (2020) compiled recent publications focused on AS studies in different tree species at various stages of development and in response to various stresses. The group, highlighted major contributions in the study of AS in woody species that have been focused on development and stress-responses, including fruit ripening (Mica et al., 2010; Gupta et al., 2015), flower morphogenesis (Ai et al., 2012), wood formation (Xu et al., 2014), drought stress (Guo et al., 2017; Ding et al., 2020), and cold stress (Xiao and Nassuth, 2006; Rahman et al., 2014; Li et al., 2020). Strategies to advance in the functional analysis of AS in woody species have been proposed (Chen et al., 2020). These are some examples of the applicability of these methods in angiosperms: phylogenetic analysis and spatial expression analysis to unravel functional conserved genes and *cis*-elements involved in AS (Li et al., 2017; Liu et al., 2017); identification of regulatory genes in signaling cascades that exhibit AS in specific stress-depend patterns (Li Z. et al., 2019) specifically induced by temperature stress (cold and heat) (Palusa et al., 2007). The potential of these methods relies entirely on the completeness of the genome-wide references, the accuracy of the genome annotations and proper bioinformatics tools. Therefore, we are still far from being able to use them in gymnosperms. For some woody species, there is already a substantial amount of transcriptome data delivered from methods focused on measuring gene expression (e.g., RNA-seq). Other methods (e.g., ATAC-seq, DNase- and MNase- seq) that have the potential to provide information about accessible DNA and its correlation with active regions of the genome involved in adaptation to climate changes could be applied to conifers. In parallel with the advance in the applications of these methodologies, new bioinformatics tools have become available to identify APA (Ha et al., 2018) and allele-specific AS from large populations (Demirdjian et al., 2020) in transcriptome data, however, they need to be implemented for analysis in gymnosperms.

### Epigenetics and Stress Memory

In the cells, transcription selects and doses gene activity by following the DNA blueprint while chromatin provides the platform that is necessary for the interaction between transcription factors, co-regulators and polymerase activity. Despite the terms epigenetics has been used for more than 50 years, the nature of the concept has jointly evolved with the advances of the omics era and has been recently reevaluated in the light of its impact on stress responses in both animals and plants. The epigenome can be defined as all the chemical compounds that are “on or attached” to DNA and cause modification in its function without altering the sequence (Riggs et al., 1996). An increasing number of publications provide evidence of mitotically and/or meiotically heritable traits in gene function attributed to epigenetic mechanisms and associated with changes in the environmental conditions (Vyse et al., 2020). The environmental cues experienced by parent trees during the reproductive process can affect growth and adaptive traits of their progeny as a result of epigenetic changes. In forest trees, this phenomenon was initially described as seed orchard “aftereffects” (Bjørnstad, 1981; Johnsen, 1989a,b), after being observed in conifers when northern selections of Norway spruce [*Picea abies* (L.) Karst.] were moved to a southern seed orchard location in order to enhance seed production. Bud set in full-sib progeny produced by the cloned northern selections located in the southern orchard (lat. 58° N) environment were delayed up to 3 weeks compared to their half-sibs from the same mother trees growing in the northern (63° N – 66° N) natural stand (Bjørnstad, 1981). The orchard progeny were also more susceptible to frost damage compared to their natural-stand counterparts when subjected to low temperatures in a phytotron (Johnsen, 1989a). When field tested on a northern site, the phenology and extended height growth of the orchard progeny resembled that of southern ecotypes (Johnsen, 1989b). Similar epigenetic effects have since been observed in other conifer species, including white spruce (*Picea glauca*) (Stoehr et al., 1998), Scots pine (*Pinus sylvestris*) (Dormling and Johnsen, 1992), and *Larix* spp. (Greenwood and Hutchison, 1996).

It follows that an adaptive epigenetic memory may have important implications for seed production sites in the southeastern United States. According to Schmidtling (1987), locating southern pine seed orchards south of the origin of selected trees can result in improved flowering and seed production. The hypothesis that the parental environment in the orchard could alter progeny performance of southern pines in the Southeast was tested in shortleaf pine (*Pinus echinata* Mill.). Ramets of 22 shortleaf pine clones were established in two diverse environments, one located in central Arkansas, United States (34.6° N), near the source of the ortets, and the second site located in south Mississippi (30.5° N) (Schmidtling and Hipkins, 2004). Thirteen identical controlled crosses (full-sib families) were made at each location, and the resulting seedlings were planted at the two locations where the seed was produced. After four growing seasons, trees from seed produced in the two environments differed significantly in height, with families produced in the warmer (southern) environment generally being taller at both planting locations. The reproductive environment × family interaction was also significant, indicating that the effect depended upon the genetic background of the parent trees. By age 9 years, the reproductive environment effect was no longer statistically significant (Schmidtling and Hipkins, 2004). In the study by Schmidtling and Hipkins (2004), there was no reference of cold damage at the time of planting or during the immediate winters that followed, but major ice storm damage in the northern planting was reported following the eighth growing season. The ice-damage was quantified using a six point (0–5) categorical scoring system. The mean ice-damage score for trees produced in the northern reproductive environment was 2.38 vs 2.62 for those produced in the southern reproductive environment. The difference, though small, was statistically significant (*P* = 0.018). The authors concluded that after-effects of reproductive environment do exist in shortleaf pine for growth and adaptive traits but are not as clear-cut nor at the same scale as that reported by Johnsen (1989a, b) in the Norway spruce studies. According to Schmidtling and Hipkins (2004), one explanation is that the north–south distance between the shortleaf seed sources amounts to only 4.1 degrees latitude rather than the 5–8 degrees latitude difference in the Norway spruce studies. In the case of loblolly pine, most seed orchards are located in the central and southern portion of the species distribution. A similar level of epigenetic effects to those reported for shortleaf pine might be expected for loblolly pine, given the vast majority of planted areas of loblolly pine in the southeastern United States are within 5 degrees latitude of the seed orchard locations.

## Working Toward Next-Generation Breeding

### Phenomics and High-Throughput Phenotyping

The current major goal of biological sciences is to generate functional models that integrate genome and phenome, allowing a multidimensional knowledge of the biology of organisms and the prediction of how they will behave under future scenarios. Several emerging global technologies (e.g., genomics, proteomics, metabolomics) are identifying and measuring changes in the molecular components under a broad set of experimental conditions including several abiotic and biotic stresses. On the other hand, next-generation phenomics measures changes in the physical attributes on a genome-wide scale that an organism overcomes during its lifespan. Both data and analytical pipelines have grown exponentially by the application of high-throughput systems. Advances in these areas are creating the next-generation breeding techniques that will reshape the way we have done agriculture until now (revised in Chen et al., 2014; Esposito et al., 2019; Yang et al., 2020). Recently, molecular breeding techniques that center the attention in DNA-features that are tightly linked to phenotypic traits, seem to be growing faster than the methods that allow the characterization of those traits in the field, and that are key components for measuring and validation of the level of improvement during selection.

The introduction of a number of highly user−friendly (and portable) chlorophyll fluorometers as well as more sophisticated high throughput hyperspectral imaging have enhanced not only the accuracy of chlorophyll measurements but also the implementation of large phenotyping platforms with the automatic control and data analysis system, which allows performing parallel studies of a large amount of plants during long cycles of growth under constant monitoring and different environments (Yao et al., 2018). Light energy absorbed by chlorophyll molecules in a leaf can undergo one of three fates: it can be used to drive photosynthesis (photochemistry), excess energy can be dissipated as heat or it can be re−emitted as light, which is observed as chlorophyll fluorescence (Maxwell and Johnson, 2000). The radiation reflected from a surface as a function of the wavelength is called the spectral signature. The spectral signature of a plant is related to its biochemical constituents, of which leaf chlorophyll content is both sensitive to environmental conditions and has a strong influence on leaf optical properties and canopy albedo (Blackburn and Pitman, 1999; Baltzer and Thomas, 2005). Therefore, chlorophyll fluorescence provides a powerful tool to infer information related to the photosynthetic apparatus and its associated metabolism (Humplík et al., 2015; Cen et al., 2017). Hyperspectral data can be used to detect subtle features across the entire visible and near-infrared regions of the electromagnetic spectrum, which correlate especially well with major leaf pigments such as the leaf chlorophyll content (Zhang et al., 2008). This could be particularly helpful under current climate change because healthy and stress vegetation present difference reflectance features in the green peak and along the red edge due to changes in pigment levels (Rock et al., 1988; Belanger et al., 1995; Gitelson and Merzlyak, 1996). Some of the commonly used physiological indicators to assess plant water conditions include stomatal conductance (Zhou et al., 2013), leaf water potential (Dzikiti et al., 2010; Zhou et al., 2013), leaf equivalent water thickness (Danson et al., 1992), and relative water content (Hunt et al., 1987; Maki et al., 2004). By obtaining complete reflectance curves at high spatial and spectral resolutions, specific plant chemical traits, including leaf water content, can be modeled and extracted at fairly high accuracies and used as phenotypes to describe plant stress (Lowe et al., 2017).

During the last 20 years researchers have advanced in the development of high throughput plant phenomics studies in controlled environments like growth chambers and greenhouses by the implementation of imaging systems, pipelines for analysis and interpretation models that would enable the use of genotypic data and novel gene discovery in a more efficient way, moving these systems toward a real optimization of plant breeding. There are limitations for predicting the performance of commercial plant varieties under field conditions based on plant phenotyping in controlled environments, but these systems are particularly useful in studying how plant phenotypes vary among different genotypes in response to controlled stress conditions. Furthermore, the control over different parameters serve to study the interactions between genetic and environmental factors that can produce unanticipated phenotypic responses (Ge et al., 2016). Previous studies have used hyperspectral imaging to determine and quantify early signs of drought stress of maize (Ge et al., 2016; Pandey et al., 2017) and cereals (Behmann et al., 2014); however, there remains a lack of targeted experiments for measuring drought indicators in loblolly pine. In pines, the content of pigments could be markedly affected by growing conditions, plant age, needle age and origin of the seed source (Linder, 1972). Because hyperspectral imaging captures spatial and spectral information that is usually processed by traditional automated image processing tools and interactive analytical approaches derived from spectroscopy, the first step in the investigation and improvement of these methods to assess drought stress in the greenhouse, is to explore more approaches to handle the plant material in different phenotypic stages and to create models that ultimately will help to increase the sensitivity and accuracy of the detection of early signs.

### Integrating Microbiome Effects

Multiple reports have described significant effects on plant drought response of growing plants in soil from a region with less precipitation than is typical of the natural range of the species in question (O’Brien et al., 2018; Allsup and Lankau, 2019; Ulrich et al., 2020). O’Brien et al. (2018) tested effects of different soil microbiota and water availability levels on three species: *Trifolium stellatum*, *Lagarus ovatus*, and *Sisymbrium erysimoides*, a legume, a grass, and a forb respectively. Under conditions of limited water availability, the grass and the forb species both produced more biomass when grown in soil from a drier site than in soil from a region with higher average precipitation. Allsup and Lankau (2019) grew seedlings of *Ostrya virginiana* (American hop hornbeam) and *Betula nigra* (river birch), both angiosperm tree species native to eastern North America, in sterilized soil from different regions inoculated with soil microbiome samples from different regions. They found that microbial inoculum from regions with lower precipitation supported greater seedling biomass production under water-limited growth conditions than the inoculum from regions with higher precipitation, regardless of the soil source. Ulrich et al. (2020) compared chlorophyll fluorescence parameters among loblolly pine families from the western (drier) and eastern (wetter) extremes of the natural range of loblolly pine, for both control seedlings grown in sterile sand and treated seedlings grown in sterile sand inoculated with soil from New Mexico, which is west of, and much drier than, the natural range of loblolly pine. They reported that control and inoculated seedlings from the eastern “wet” family showed no difference in the rate of decline of the Fv/Fm ratio of chlorophyll fluorescence in a time series of measurements taken over 4 weeks after they stopped providing water to the seedlings. In contrast, control seedlings of the western “dry” family showed a slower rate of decline in the Fv/Fm ratio than inoculated seedlings of the “dry” family, suggesting that the inoculated soil had a negative effect on the “dry” family but not on the “wet” family. Both Ulrich et al. (2020) and Allsup and Lankau (2019) speculated that the use of soil inocula from regions drier than the climate region from which the experimental plants were drawn contributed to the observed effects of inoculation in their respective studies. An alternative hypothesis is that the effects are due to the presence of novel species of soil microorganisms to which the plants had not been previously exposed.

A meta-analysis of data from 126 papers (Karst et al., 2018) presented evidence that interactions between 59 host tree species and 52 genera of ectomycorrhizal fungi tend to have positive effects on average, and the degree of positive effect was generally larger when the host and fungal species used came from allopatric rather than sympatric ranges. In other words, the response to inoculation tended to be larger when the host plant and fungal inoculum came from regions farther apart. This effect was detected both when using only data from studies with species of pines (the genus with greatest representation among all the studies examined), or using data from all studies except those on pines, so the results are not driven by any unique response of the most-studied plant genus. The question of whether this effect might account for some fraction of the differences in plant drought responses attributed to inoculation with soil microbiota from drier regions (O’Brien et al., 2018; Allsup and Lankau, 2019; Ulrich et al., 2020) remains to be determined. Future experiments to test the effects of inoculation with soil microbiota from dry regions on plant drought responses should include control inoculations with soil microbiota from regions equally distant from the native range of the host plant, but with similar water availability, to test the hypothesis that specific effects on drought responses are related to the water availability in the region from which the microbial inoculum was obtained, rather than simply the distance from the host plant range.

## Future Directions

**FIGURE 3:**
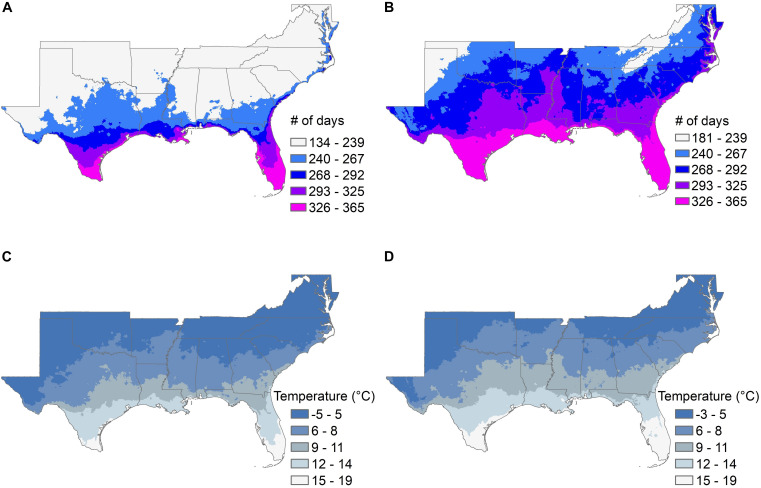
The average number of frost-free days in the growing season **(A,B)** and the average March minimum temperature **(C,D)** are shown for the southeastern United States for the 30-year historical record from 1976 to 2005 **(A,C)**, and from 2051 to 2080 **(B,D)**. The color scales in the legends are the same for both time periods, but the lowest category for frost-free days and for average March minimum temperatures are slightly different because the lower limits are higher in the projections for 2051 to 2080.

**FIGURE 4:**
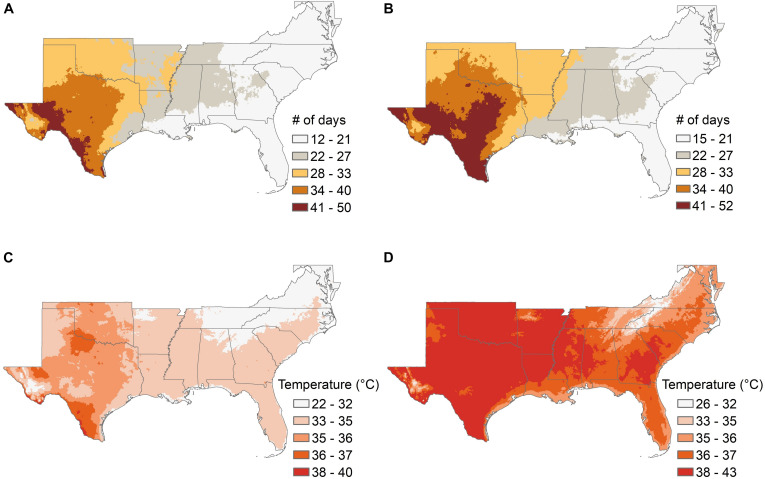
The average number of days without precipitation during summer months **(A,B)** and the average July maximum temperatures **(C,D)** are shown for the southeastern United States for the 30-year historical record from 1976 to 2005, and from ensemble climate model projections using the RCP 8.5 scenario for the period from 2051 to 2080. The boundaries of the lowest and highest categories for both pairs of maps are slightly different between the maps showing the historical record and those showing projected future conditions because the minimum and maximum values increased in the future projections, but the color scales are the same for both time periods.

Climate models suggest that the southeastern United States is likely to show significant changes over the next 50 years in the length of the frost-free growing season and the average late-winter (March) minimum temperature (Figure 3 and Supplementary Table 1), and in the average length of summer “dry periods” between rainfall events and the average July maximum temperatures (Figure 4 and Supplementary Table 1). The current projected duration of a cycle of loblolly pine breeding is 14 years (Isik and McKeand, 2019), so in the absence of revolutionary changes, two breeding cycles can be completed for loblolly pine by 2050. The 30-year time period used for Figures 3, 4 climate factors is typically sufficient for a loblolly pine plantation to grow from establishment to harvest, so trees planted by 2051 will generally be harvested by 2080. Projected differences in the number of frost-free days and March minimum temperatures (Figure 3) do not highlight obvious risks for pollination, seedling production, or plantation growth, although these plots do not show the risk of “weather whiplash.” Projected changes in the maximum number of days between rainfall events and in the average maximum July temperature (Figure 4) do suggest that tolerance (resistance, resilience, or both) of higher temperatures and lower water availability will be a valuable trait for loblolly pine planting stock.

Intra-specific genetic variation for drought tolerance has been observed in many species, including genera such as *Picea*, *Pinus*, *Abies*, and *Cryptomeria*. Several early studies with loblolly pine reported genetic variation both among loblolly pine provenances and among families within provenances (Zobel, 1955; Cech and Goddard, 1957). The observation of existing genetic variation for drought tolerance suggests that breeders could select for improved drought responses, either resistance or resilience or both, provided that cost-effective methods for testing could be developed to allow collection of appropriate phenotypic data for enough individuals. Breeders working to improve loblolly pine often are interested in comparing the performance of dozens to hundreds of different individuals, and depending on the nature of experimental designs used, this can mean measuring phenotypes of hundreds to thousands of plants. By reviewing publications about physiological responses to water and cold stress, we identified some studies that would provide valuable information about how loblolly pine is adapting to changes in climate at the cellular and physiological levels. This includes research on ABA-stomatal control and application of micro-computed tomography to detect embolism and conduct refilling. However, these detailed physiological assessments are often impractical for such large numbers of individual plants, so past efforts to measure drought responses in loblolly pine for breeding purposes have often relied on common-garden field trials where seedlings grown from seed collected in different parts of the natural range of loblolly pine are planted together in trials, often in multiple locations. A recent analysis that looked for time-course trends over the last 20 years in six top impact factor journals, revealed that the fraction of papers that represented the interaction between the topics “omics” x ecophysiology represented <4% on average compared with papers on both terms in 2017 (Flexas and Gago, 2018). This could be an opportunity for ecophysiologists and omics-researchers to look for multidisciplinary projects and focus on the improvement of robust predictive models for breeding.

Two key questions for breeders are what phenotypes to measure, and which trees to select. The answer will be different for different species, because the sensitivity to drought and the hazard from drought will vary across regions and species. For loblolly pine in the southeastern United States, the initial stage of plantation establishment is particularly important in terms of drought. The first year after seedlings are planted in the field is the most critical time in terms of water availability, because the root systems are still small and unable to reach water in deeper soil horizons. Selection for the ability of pine seedlings to survive a period of limited water availability during the first year could reduce the risk of plantation failure, and should also be a relatively easy trait to measure under controlled conditions. An additional advantage of working with seedlings at this age may be that the physiological or genetic mechanisms of resistance need not be identified in advance, provided that screening methods mimic as closely as possible the kind of drought stress to which resistance is desired. Projections from ensemble climate models (Figure 4B) suggest that the ability to survive a period of 3–4 weeks without rainfall during the first summer after planting is likely to be a valuable trait to increase the likelihood of successful plantation establishment in much of the southeastern United States by mid-century. Seedlings can be exposed to such stresses under controlled conditions now, either in greenhouse studies with controlled delivery of water (Marchin et al., 2020), or by field planting seedlings in regions with little to no summer rainfall and providing supplemental irrigation during spring growth and then withdrawing irrigation to impose the desired drought stress during the summer. The latter method could also have the additional feature of imposing higher summer temperatures than are currently common in the natural range of loblolly pine, but may become common in the future (Figure 4D).

Integrative studies that combine surveys of genetic variation across the natural range with analyses of gene expression, high-throughput phenotyping data, and analyses of replicated test plantings derived either from experimental crosses or from accessions collected from natural populations, have provided new insights into mechanisms of drought tolerance and other complex phenotypes in some non-model species (Lovell et al., 2018; Weighill et al., 2019a,b). Some efforts toward this kind of analysis have been made in conifers (De La Torre et al., 2019; Lu et al., 2019a,b; Mahony et al., 2020), but they are constrained by the lack of high-quality sequenced genomes and annotation for most conifers, as previously discussed. The current (v2.01) loblolly pine assembly allowed mapping of fewer than half the SNPs associated with polymorphisms in a recent genome-wide association study onto scaffolds anchored to linkage groups in the genetic map (De La Torre et al., 2019). More than half of the trait-associated SNPs were in non-coding regions, and the relationship between those variants and any genes whose expression levels or tissue-specificity might be affected by the variant locus cannot be determined given the current fragmented assembly (Zimin et al., 2017).

Genomic approaches to understand and improve drought responses in trees have been reviewed (Hamanishi and Campbell, 2011; Moran et al., 2017; Isabel et al., 2020), and additional reports of association studies in conifers have been published since then (Trujillo-Moya et al., 2018; De La Torre et al., 2019; Lu et al., 2019b; Mahony et al., 2020). Associations can be identified between genotypes and phenotypes, an approach common in crop and model plant species as well as animal genetics and human biomedical research, or between genotypes and environmental variables for samples collected from across the range of a species (Rellstab et al., 2015). A common theme to the results of such studies in loblolly pine has been that individual single-nucleotide polymorphisms (SNPs) associated with drought response or environmental variables related to water availability or aridity typically explain a relatively small proportion of the observed variation. Many of the SNPs associated with phenotypic or environmental variation also show low minor-allele frequency in the population, suggesting that many such loci would have to be genotyped in many individual trees in order to have enough power to model phenotypic variation for breeding purposes. Multivariate methods that analyze multiple SNP alleles (sometimes grouped by functional categories such as the genes in which those SNPs occur) and multiple phenotypes in parallel have shown improved power to detect associations of genotypes with environmental variables (Rellstab et al., 2015; Forester et al., 2018) and with phenotypic variation (Kaakinen et al., 2017; Luo et al., 2020). Genomic selection is another approach to parallel analysis of many SNP loci with phenotypic information, and considerable interest has been shown in applying this method to forest tree breeding (reviewed by Lebedev et al., 2020).

Most conifer breeding programs work with families of progeny as the genetic entries in field tests, because inbred lines are typically not available. The most advanced breeding programs in loblolly pine are only five to eight generations from trees selected from wild populations, but pedigree records showing the relationships across multiple generations are available for many individuals in the advanced-generation breeding populations (Isik and McKeand, 2019). These family-based structured breeding populations will be suitable for haplotype-based analyses, which have been shown to have greater sensitivity for detection of the genetic basis of complex clinical phenotypes in human populations (Howard et al., 2017). A key requirement for such analyses, however, is the ability to detect “haplotype blocks,” or groups of variant alleles that are on the same chromosome homolog and are inherited together through multiple generations. Biomedical researchers have been working for the past two decades to collect data on the haplotype structure of the human genome (Gabriel et al., 2002; The International HapMap Consortium, 2003; Altshuler et al., 2005). Across the human population as a whole, these haplotype blocks are an imperfect representation of linkage disequilibrium among SNP alleles (Wall and Pritchard, 2003), but within groups of individuals that are related by descent from a common ancestor, population haplotype data can be used to detect haplotypes that are “identical-by-descent” (IBD) from a common ancestor (Browning, 2008; Gusev et al., 2011).

Patterns of linkage disequilibrium in the loblolly pine genome are very different from those found in human populations (Brown et al., 2004; Lu et al., 2016; Acosta et al., 2019), so a population-based “haplotype map” will only be possible for a group of related trees descended from a relatively small number of founding parents. This strategy is similar to that used in Multi-parent Advanced Generation InterCross (MAGIC) populations in crop breeding and model plant and animal studies (Cavanagh et al., 2008; Kover et al., 2009; Threadgill et al., 2011). When a high-quality genome assembly and annotation are available for loblolly pine, long-read DNA sequencing methods (Oxford Nanopore or Pacific Biosciences) can be used to detect long-range linkages among SNP alleles to identify parental haplotypes, followed by SNP array-based or sequence-based genotyping of progeny to impute the entire complexity of the parental genome sequences to the progeny. This approach would enable a combination of haplotype-based methods for associating phenotypic variation to specific chromosome haplotypes with RNA-seq-based analysis of gene expression, AS, and APA sites usage to collect comprehensive genomic datasets for multivariate analysis. Conducting such analyses in parallel with several MAGIC populations, each composed of crosses among loblolly pine parents from different parts of the natural range and planted in replicated field trials in multiple environments, would provide a valuable resource for the dissection of genetic variation controlling complex phenotypes in pine. Phenotypes in such test plantings could be measured using high-throughput methods such as hyperspectral imaging for phenological differences (D’Odorico et al., 2020), acoustic stress wave and drill resistance methods for wood properties (Walker et al., 2019), and terrestrial LIDAR (Liang et al., 2016). Integrative analysis of large datasets with multiple phenotypes and genomic data sources would be a powerful tool in helping tree breeders better understand and work with the natural genetic variation so abundant in loblolly pine populations. Accelerating breeding progress will be essential for meeting social needs for forest products in the second half of the 21st century.

## Author Contributions

RW and LM-R conceived the idea of the review and prepared the initial outline. LM-R contributed Figure 2. RW, LM-R, and KP gathered the literature for all sections. GS contributed the analysis of climate change models and Figures 1, 3, 4. All authors contributed to revising and editing the draft sections, and approved the content of the final manuscript.

## Conflict of Interest

KP is Director of the Cooperative Tree Improvement Program at NC State University, a public-private partnership founded in 1956 that receives funding from stakeholders in the forestry sector including landowners, seed producers, seedling nurseries, and timberland management organizations. The remaining authors declare that the research was conducted in the absence of any commercial or financial relationships that could be construed as a potential conflict of interest.
