# Supplementary material for: Breeding for Climate Change Resilience: A Case Study of Loblolly Pine (Pinus taeda L.) in North America
Source: Front Plant Sci. 2021 Apr 30;12:606908. doi: 10.3389/fpls.2021.606908 (PMC8119900; doi:10.3389/fpls.2021.606908)
Supplement: Supplementary file 1 [file Table_1.DOCX]

***Supplementary Material***

**Supplementary table 1.**

Historical and future climate conditions across the southeastern US were characterized by the average March minimum temperature, July maximum temperature, number of dry days, and number of frost-free days (Table S1). Historical and future average temperatures were estimated using 20 statistically downscaled Global Climate Models (GCMs) from the Multivariate Adaptive Constructed Analogs datasets version 2 (MACAv2-METDATA, Abatzoglou and Brown 2011). This dataset is bias-corrected to match the spatial patterns and statistical properties of observed conditions. Historical estimates and future projections of consecutive dry days and frost-free days were obtained from the U.S. Department of Agriculture Forest Service National Forest Climate Change Maps project (USDA Forest Service 2019). Future climate conditions follow an increasing atmospheric greenhouse gas emissions scenario (Representative Concentration Pathway 8.5, van Vuuren et al. 2011). These datasets spatial resolution is approximately 4 km. The digital representation of loblolly pine (*Pinus taeda L.*) natural range was obtained from the USGS tree species range maps (Little 1971, USGS 1999).

Table S1. Historical and future climate data source and description.

| Data | Description | Source | Access |
| --- | --- | --- | --- |
| Natural range | Natural range of loblolly pine (*Pinus taeda L.*) | Little 1971, USGS 1999 | [Retrieved from https://web.archive.org/web/20170127093428/https:/gec.cr.usgs.gov/data/little/, accessed June 2020](https://climate.northwestknowledge.net/MACA/data_portal.php) |
| Number of frost-free days | Average length of the frost-free seasons, for historical (1976-2005) and future (2071-2090, RCP 8.5) time periods were estimated from 20 statistically downscaled GCMs using the MACA datasets version 2 (MACAv2-METDATA) of the CMIP5 | USDA Forest Service 2019 | [Retrieved from https://data.fs.usda.gov/geodata/rastergateway/OSC/climate.php, accessed June 2020](https://climate.northwestknowledge.net/MACA/data_portal.php) |
| Average winter minimum temperature | Historical (1976-2005) and future (2051-2080, RCP 8.5) climate averages of March minimum temperatures were estimated from 20 statistically downscaled GCMs using the MACA datasets version 2 (MACAv2-METDATA) of the CMIP5 | Abatzoglou and Brown 2011 | [Retrieved from https://climate.northwestknowledge.net/MACA/data_portal.php, accessed June 2020](https://climate.northwestknowledge.net/MACA/data_portal.php) |
| Number of dry days | Average length of consecutive days without precipitation during summer, for historical (1976-2005) and future (2071-2090, RCP 8.5) time periods were estimated from 20 statistically downscaled GCMs using the MACA datasets version 2 (MACAv2-METDATA) of the CMIP5 | USDA Forest Service 2019 | [Retrieved from https://data.fs.usda.gov/geodata/rastergateway/OSC/climate.php, accessed June 2020](https://climate.northwestknowledge.net/MACA/data_portal.php) |
| Average summer maximum temperature | Historical (1976-2005) and future (2051-2080, RCP 8.5) climate averages of July maximum temperatures were estimated from 20 statistically downscaled GCMs using the MACA datasets version 2 (MACAv2-METDATA) of the CMIP5 | Abatzoglou and Brown 2011 | Retrieved from https://climate.northwestknowledge.net/MACA/data_portal.php, accessed on June 2020 |

GCM = Global Climate Models; RCP = Representative Concentration Pathway; MACA = Multivariate Adaptive Constructed Analogs; CMIP5 = Coupled Model Intercomparison Project phase 5.

References

Abatzoglou, J.T., and Brown T.J. (2011). A comparison of statistical downscaling methods suited for wildfire applications. Int. J. Climatol., 32, 772-780, 10.1002/joc.2312.

Little, E.L., Jr., (1971). Atlas of United States trees, volume 1, conifers and important hardwoods. U.S. Department of Agriculture Miscellaneous Publication, 1146, 9 p., 200 maps.

U.S. Department of Agriculture Forest Service (2019). Historical and projected climate datasets. https://data.fs.usda.gov/geodata/rastergateway/OSC/climate.php [Accessed June, 2020].

U.S. Geological Survey. (1999). Digital representation of "Atlas of United States Trees" by Elbert L. Little, Jr. https://web.archive.org/web/20170127093428/https:/gec.cr.usgs.gov/data/little/ [Accessed June, 2020].
